# An annotated checklist of Microweiseinae and Sticholotidini of Iran (Coleoptera, Coccinellidae)

**DOI:** 10.3897/zookeys.587.8056

**Published:** 2016-05-10

**Authors:** Amir Biranvand, Oldřich Nedvěd, Wioletta Tomaszewska, Claudio Canepari, Jahanshir Shakarami, Lida Fekrat, Mehdi Zare Khormizi

**Affiliations:** 1 Department of Entomology, College of Agricultural Sciences, Shiraz Branch, Islamic Azad University, Shiraz, Iran; 2 Faculty of Science, University of South Bohemia, Branišovská 1760, CZ-37005 ČeskéBudějovice, Czech Republic; 3 Institute of Entomology, Biology Centre, Branišovská 31, 37005 ČeskéBudějovice, Czech Republic; 4 Museum and Institute of Zoology, Polish Academy of Sciences, Warszawa, Poland; 5 Via Venezia 1, I-20097 San Donato Milanese, Milan, Italy; 6 Plant Protection Department, Lorestan University, Agricultural faculty, Khorramabad, Iran; 7 Department of Plant Protection, Faculty of Agriculture, Ferdowsi University of Mashhad, Mashhad, Iran

**Keywords:** Coccinelloidea, distribution, host plants, Microweiseinae, prey species, Sticholotidini, updated checklist

## Abstract

An updated checklist of the Coccinellidae species of the former subfamily Sticholotidinae recorded from Iran is provided. Eleven species are reported: two species classified presently in the subfamily Microweiseinae (in the genera *Paracoelopterus* Normand, 1936 and *Serangium* Blackburn, 1889), and nine species classified in the tribe Sticholotidini of the subfamily Coccinellinae (in the genera *Coelopterus* Mulsant & Rey, 1852 and *Pharoscymnus* Bedel, 1906). *Pharoscymnus smirnovi* Dobzhansky, 1927 is removed from the list of the Coccinellidae of Iran. Distribution of species in Iranian provinces is presented. Data concerning their host plants along with their prey species are also included when known. Morphological features of two rarely collected and poorly known species of Iranian fauna, *Pharoscymnus brunneosignatus* Mader, 1949 and *Pharoscymnus pharoides* (Marseul, 1868) are diagnosed and illustrated.

## Introduction

The family Coccinellidae with approximately 6000 species and 360 genera was classified until recently in the superfamily Cucujoidea (Coleoptera, Polyphaga) and placed in the Cerylonid Series, a derived clade formed by Cerylonidae and eight other families of Cucujoidea (e.g. Crowson 1955; Lord et al. 2010). The most recent molecular research by Robertson et al. (2015) revealed, however, the Cerylonid Series as monophyletic group sister to the remaining Cucujiformia, not allied with any superfamily of the Cucujiformia including the remaining Cucujoidea. For these families, Robertson et al. (2015) established a new superfamily Coccinelloidea.

Most of the standard classifications of Coccinellidae (Sasaji1968, 1971, Gordon 1985, Kovář 1996, Vandenberg 2002) recognized six or seven subfamilies (Coccinellinae, Coccidulinae, Scymninae, Chilocorinae, Epilachninae, Sticholotidinae and, sometimes, Ortaliinae) with numerous tribes within each subfamily. Ślipiński (2007) found these classifications as phylogenetically unacceptable and argued the basal split of Coccinellidae into two subfamilies Microweiseinae and Coccinellinae comprising all the remaining coccinellid groups.

This split of the family was confirmed by subsequent molecular and combined molecular and morphological research (Robertson et al. 2008; Giorgi et al. 2009, Seago et al. 2011, Robertson et al. 2015). But Nedvěd and Kovář (2012) incorporated some results of recently published molecular and morphological research, and proposed nine subfamilies and 42 tribes.

Small and the least apparent members of Coccinellidae were historically placed in the subfamily Sticholotidinae described by Weise (1901) and redefined by Sasaji (1968, 1971). Sticholotidinae (*sensu* Sasaji 1968) contained four tribes: Sticholotidini Weise, Shirozuellini Sasaji, Serangiini Blackwelder and Sukunahikonini Kamiya (Vandenberg and Perez–Gelabert 2007) and was defined primarily by the presence of a narrow and apically pointed terminal maxillary palpomere and a narrow junction between mentum and submentum. However, subsequently included tribes Limnichopharini Miyatake, Argentipilosini Gordon and Almeida, Plotinini Miyatake, Cephaloscymnini Miyatake and Carinodulini Gordon, Pakaluk and Ślipiński, with the terminal maxillary palpomere paralel sided, distally expanded or even securiform, made this group taxonomically heterogenous.

Kovář (1996) in a comprehensive classification of Coccinellidae divided Sticholotidinae into ten tribes without providing any basis for the monophyly of this subfamily. It was later recognized as polyphyletic group (Duverger 2003, Ślipiński and Tomaszewska 2005, Vandenberg and Perez-Gelabert 2007). Ślipiński (2007) proposed the formal classification of Coccinellidae with Microweiseinae containing Sukunahikonini, Microweiseini, Serangiini and Carinodulini, while placed remaining tribes of the former Sticholotidinae (Shirozuellini, Limnichopharini, Argentipilosini, Cephaloscymnini, Plotinini, Sticholotidini) in a redefined subfamily Coccinellinae. Nedvěd and Kovář (2012) in their classification placed these tribes in the narrowly defined subfamily Sticholotidinae.

After the split of former Sticholotidinae, research conducted so far revealed well defined Microweiseinae. This subfamily contains now three tribes (Microweiseini including Sukunahikonini, Serangiini and Carinodulini) and is well defined by a set of morphological characters: antenna inserted in front of eyes, often separated from eyes anteriorly, antennal insertions exposed and close together, clypeus well developed and emarginate around antennal insertions, subgena with glandular openings, mandible simplified with single apical tooth and no mola; ventral mouthparts retracted causing unusual projection of genae into a frame enclosing strongly elongate maxillae and labium; male genitalia with asymmetrical tegmen (Escalona and Ślipiński 2012). The remaining tribes of former Sticholotidinae either together or most tribes separately do not form clearly defined taxonomic entities and need more study. The geniculate maxillary palps with terminal maxillary palpomere pointed, bearing long oblique sensory area and compact antenna with spindle-shaped club bearing group of short sensory setae on the terminal antennomere were listed as characters for subfamily Sticholotidinae by Nedvěd and Kovář (2012). To date, these tribes are treated as a widely conceived tribe Sticholotidini in the widely conceived subfamily Coccinellinae (Ślipiński 2007).

The recent checklist of Coccinellidae of Iran provided by Moddarres-Awal (2012) included 125 species of which only seven species belong to the subfamily Sticholotidinae *sensu* Sasaji (1968) and Kovář (1996): *Diloponis fuerschi* Yazdani & Ahmadi, 1992, *Pharoscymnus arabicus* Fürsch, 1979, *Pharoscymnus flexibilis* (Mulsant, 1853), *Pharoscymnus ovoideus* Sicard, 1929, *Pharoscymnus pharoides* Marseul, 1868, *Pharoscymnus setulosus* (Chevrolat, 1861), *Serangium montazerii* Fürsch, 1995.

The current study was inspired by a collection of the new material of species belonging to the former Sticholotidinae and was aimed to update the information on the current classification, occurrence, host plants and the prey of species of this group in Iran. Similar studies on other, more speciose, tribes of the family will follow.

## Material and methods

The study area in Iran is located in southwest of Asia in the Middle East region. More than half of the country’s land is arid or semi-arid; almost one third of the country is mountainous and a small part contains fertile plains. In winter, the temperature difference between the coldest and warmest place may exceed 50 °C. Precipitation in Iran is highly variable, from more than 2000 mm of rain a year in north to less than 15 mm in desert areas.

The arrangements of tribes, genera and species are listed alphabetically for convenience, according to classification of Seago et al. (2011). The geographical distribution, host plants and prey species are given for all the species based on literature and labels of the museum specimens examined by the first author and on personal observations of authors. The geographical distribution therein also is arranged according to the year of record publication and in alphabetical order.

Identification of *Pharoscymnus pharoides* (Marseul, 1868) was based on the original description of Smirnoff (1956a). Specific terminology used in morphology of Coccinellidae follows Ślipiński (2007) and Ślipiński and Tomaszewska (2010).

New specimens examined were collected in 2013 and 2014 in different parts of Iran, and are deposited in Plant Protection Department, Lorestan University, Agricultural faculty, Khorramabad, Iran and Gorgan University of Agricultural Sciences and Natural Resources, Iran.

## Results

This checklist includes eleven species of the Sticholotidinae *sensu lato.* According to the current classification of Coccinellidae, two species belong to the subfamily Microweiseinae (to the tribes Microweiseini and Serangiini) and nine species to the tribe Sticholotidini of the subfamily Coccinellinae. *Pharoscymnus smirnovi* Dobzhansky, 1927, which was first recorded by Zare Khormizi (2014) from Iran, was removed from the list of Iranian coccinellids after re-examination of the specimens, as they appeared to be misidentified. For *Pharoscymnus pharoides* (Marseul, 1868) new locality in Iran (Lorestan province) and new host plants (pine, walnut and hawthorn trees) are recorded.

The updated list of the species is as follows:

### Subfamilly Microweiseinae Leng, 1920

#### Tribe Microweiseini Leng, 1920

##### *Paracoelopterus* Normand, 1936

###### Paracoelopterus berytensis

Taxon classificationAnimaliaColeopteraCoccinellidae

(Weise, 1884)

Paracoelopterus berytensis (= Diloponis fuerschi Yazdani & Ahmadi, 1992)

####### General distribution.

Greece, Israel, Iran, Lebanon, Morocco, Tunisia (Kovář 2007).

####### Distribution in Iran.

Fars, Sistan and Baluchestan (Ahmadi and Yazdani 1993; Moddarres-Awal 2012).

####### Host plants and prey species in Iran.

This species has been collected from almond, ash, date palm, willow and wild pistachio as the predator of Hemiptera, Diaspididae: *Chionaspis salicis* (Linnaeus), *Lepidosaphes malicola* Borchsenius, *Melanaspis inopinata* (Leonardi), *Parlatoria blanchardi* Targioni Tozzetti, *Pistaciaspis pistaciae* Borchsenius, *Pistaciaspis pistacicola* Borchsenius, *Tecaspis asiatica* Bazarov (Moddarres-Awal 2012).

#### Tribe Serangiini Blackwelder, 1945

##### *Serangium* Blackburn, 1889

###### Serangium montazerii

Taxon classificationAnimaliaColeopteraCoccinellidae

Fürsch, 1995

####### General distribution.

France, Georgia, Israel, India, Iran, Pakistan, Syria (Kovář 2007).

####### Distribution in Iran.

Gilan, Golestan, Mazandaran, Zanjan (Fürsch 1995; Hajizadeh et al. 2003; Moddarres-Awal 2012).

####### Host plants and prey species in Iran.

This species has been collected from citrus, olive, pomegranate and *Salvia* as the predator of *Euphyllura olivina* (Costa) (Hemiptera, Psyllidae) (Hajizadeh et al. 2003; Moddarres-Awal 2012).

### Subfamilly Coccinellinae Latreille, 1807

#### Tribe Sticholotidini Weise, 1901

##### *Coelopterus* Mulsant & Rey, 1852

###### Coelopterus salinus

Taxon classificationAnimaliaColeopteraCoccinellidae

Mulsantand Rey, 1852

####### General distribution.

Somalia, Syria (Plaza 1986), Algeria, France, Italy (Sardinia), Iran, Morocco, Portugal, Spain, Tunisia (Kovář 2007), The United Arab Emirates (Raimundo et al. 2008).

####### Distribution in Iran.

Iran (Kovář 2007) – no specific distribution known.

####### Remarks.

This species is known to be present in *Salicornia* habitats periodically inundated by sea water (Canepari 2010).

##### *Pharoscymnus* Bedel, 1906

###### Pharoscymnus angohranensis

Taxon classificationAnimaliaColeopteraCoccinellidae

Duverger, 1983

####### General distribution.

Iran (Kovář 2007).

####### Distribution in Iran.

Hormozgan (Duverger 1983).

###### Pharoscymnus arabicus

Taxon classificationAnimaliaColeopteraCoccinellidae

Fürsch, 1979

####### General distribution.

Iran, Saudi Arabia, The United Arab Emirates (Kovář 2007).

####### Distribution in Iran.

Fars, Gilan (Moddarres-Awal 2012).

####### Host plants and prey species in Iran.

This species has been collected from date palm as the predator of *Parlatoria blanchardi* (Hemiptera: Diaspididiae; Yazdani 1990; Moddarres-Awal 2012).

###### Pharoscymnus brunneosignatus

Taxon classificationAnimaliaColeopteraCoccinellidae

Mader, 1949

Figure 1

####### Material examined.

Iran, North Khorasan Prov., Baba Aman (37°29'34"N 57°26'19"E), Tamarisk, iv.2013, lgt. et coll. Hamidi, det. Nedvěd and Canepari.

####### Diagnosis.

**Figure 1.**
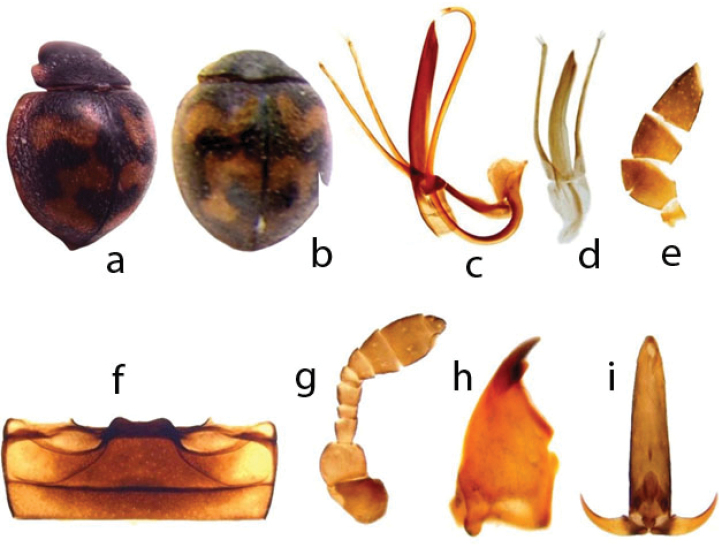
*Pharoscymnus brunneosignatus*. **a, b** dorsal view at various angles **c** aedeagus **d** tegmen **e** maxillary palp **f** abdominal ventrites 1–2 **g** antenna **h** mandible, **i** terminal tarsomere and claws.

Body length 2.1 mm. Dorsal surface black and setose with orange, transverse bands of irregular shape on elytra (Fig. 1 a, b); head, antennae and mouthparts dark brown (Fig. 1a, e, g, h). Male genitalia with penis strongly curved near base and before apex – in form of question mark (Fig. 1 c); tegminal strut about as long as basal piece, parameres slender, nearly as long as penis guide (Fig. 1c, d).

####### General distribution.

China, Mongolia (Kovář 2007), Iran (Ebrahimi et al. 2014).

####### Distribution in Iran.

North Khorasan, Khorasan Razavi (Ebrahimi et al. 2014; Nedvěd et al. unpublished).

###### Pharoscymnus fleischeri

Taxon classificationAnimaliaColeopteraCoccinellidae

(Weise, 1883)

####### General distribution.

Greece, Iran, Turkey (Kovář 2007).

####### Distribution in Iran.

Iran (Kovář 2007) – no specific distribution known.

###### Pharoscymnus flexibilis

Taxon classificationAnimaliaColeopteraCoccinellidae

(Mulsant, 1853)

####### General distribution.

Afghanistan, India, Iran, Pakistan (Kovář 2007), Oman, Yemen, The United Arab Emirates (Raimundo et al. 2008).

####### Distribution in Iran.

Fars (Moddarres-Awal 2012).

###### Pharoscymnus ovoideus

Taxon classificationAnimaliaColeopteraCoccinellidae

Sicard, 1929

####### General distribution.

Israel (Halperin et al. 1995), Iran, Jordan, Syria (Kovář 2007), Algeria, Morocco, Tunisia, The United Arab Emirates (Raimundo et al. 2008).

####### Distribution in Iran.

Fars, Gilan, Kerman, Lorestan, Tehran (Hajizadeh et al. 2003; Jafari and Kamali 2007; Abdi et al. 2012; Moddarres-Awal 2012).

####### Host plants and prey species in Iran.

This species has been collected from almond, apple, ash, citrus, conifer trees, oleander, olive, date palm, pomegranate, sloe and willow as the predator of Hemiptera, Diaspididae: *Aonidiella orientalis* (Newstead) and *Parlatoria blanchardi* (Hajizadeh et al. 2003, Jafari and Kamali 2007; Abdi et al. 2012; Moddarres-Awal 2012).

####### Remarks.

This ladybird is one of the most important predators of scale insects, including *Parlatoria blanchardi*, on palm trees (Smirnoff 1956b). This species was imported from Iran to France; after rearing, it was used against *Parlatoria blanchardi* in mixed fruit groves of Moritani in 1967 (Iperti 1970).

###### Pharoscymnus pharoides

Taxon classificationAnimaliaColeopteraCoccinellidae

(Marseul, 1868)

Figure 2 a–h

####### Material examined.

3 females, 4 males, Iran, Lorestan Prov., Azna Mmyl (33°23'00"N 48°36'05"E), on hawthorn, pine, walnut, iii.2013, lgt. et coll. Biranvand, det. Canepari.

####### Diagnosis.

**Figure 2.**
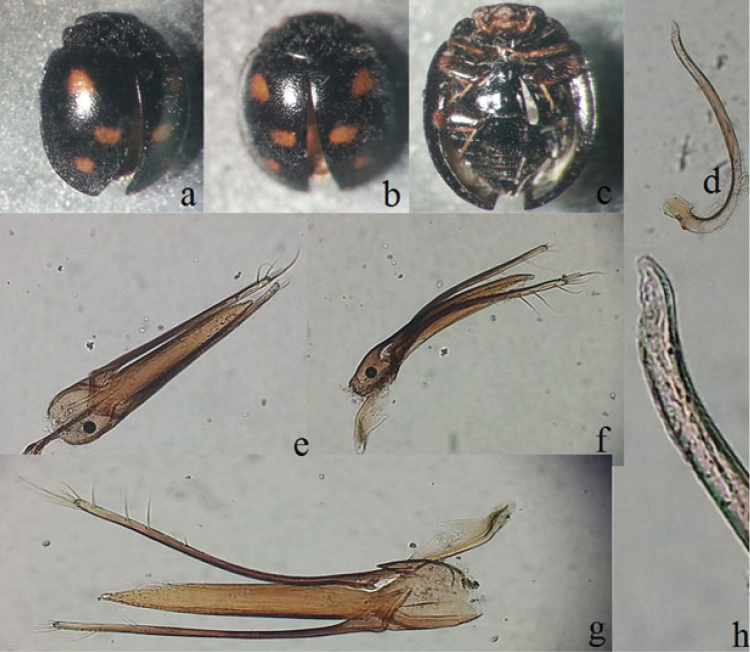
*Pharoscymnus pharoides*. **a, b** dorsal view at various angles **c** ventral view **d** penis **e–g** tegmen at various angles **h** tip of penis.

Body length 1.9 mm. Dorsal surface black and setose with three pairs of orange spots on elytra; head, antennae and mouthparts dark brown; eyes completely visible from dorsal view; coxa, trochanter and basal part of femur black, distal part of femur, tibia and tarsus dark brown (Fig. 2 a–c). Male genitalia with penis weakly curved near base (Fig. 2 d, h); tegminal strut about as long as basal piece, parameres slender and distinctly longer than penis guide (Fig. 2 e–g).

####### General distribution.

Egypt, Iran, Israel, Libya, Syria, Saudi Arabia, Turkey (Kovář 2007).

####### Distribution in Iran.

Chaharmahal and Bakhtiari, Fars (Bagheri and Mosadegh 1997, Moddarres-Awal 2012), Lorestan (current study).

####### Host plants and prey species in Iran.

This species has been collected from almond and oak (Bagheri and Mosadegh 1997), and recently from hawthorn, pine, and walnut (current study).

####### Remarks.

This species was reported by Erler and Tunc (2001) on *Olea europaea* as a predator of *Lineaspis riccae* (Targioni Tozzetti).

###### Pharoscymnus setulosus

Taxon classificationAnimaliaColeopteraCoccinellidae

(Chevrolat, 1861)

####### General distribution.

Algeria, Egypt, Iran, Israel, Jordan, Libya, Morocco, Saudi Arabia, Spain, Tunisia, The United Arab Emirates (Kovář 2007).

####### Distribution in Iran.

Fars (Moddarres-Awal 2012).

####### Host plants and prey species in Iran.

This species has been collected from date palm as the predator of *Parlatoria blanchardi* (Hemiptera, Diaspididae; Yazdani 1990; Moddarres-Awal 2012).

## Conclusion

Species of Microweiseinae and Sticholotidini from Iran belong to four genera. Eight of a total of eleven species belong to the Sticholotidini genus *Pharoscymnus*. For two species, no details are known about their distribution in Iran. Fars is the best investigated province of Iran with six known species belonging to the investigated groups of ladybirds; Gilan and Lorestan have three and two known species respectively, and the other provinces have only a single species each. Most of these species have western Palaearctic or Mediterranean distribution in general, but a few species extend to India or China.

Host plants in Iran were recorded for six species. Three species were found on both almond and date palm, two species on ash, citrus, olive and pomegranate. Prey species, always scale insects, were recorded for five of the eleven listed ladybird species. For four species, *Parlatoria blanchardi* was the single prey or one of the prey species.

## Supplementary Material

XML Treatment for Paracoelopterus berytensis

XML Treatment for Serangium montazerii

XML Treatment for Coelopterus salinus

XML Treatment for Pharoscymnus angohranensis

XML Treatment for Pharoscymnus arabicus

XML Treatment for Pharoscymnus brunneosignatus

XML Treatment for Pharoscymnus fleischeri

XML Treatment for Pharoscymnus flexibilis

XML Treatment for Pharoscymnus ovoideus

XML Treatment for Pharoscymnus pharoides

XML Treatment for Pharoscymnus setulosus
